# Research Advances in the High-Value Utilization of Peanut Meal Resources and Its Hydrolysates: A Review

**DOI:** 10.3390/molecules28196862

**Published:** 2023-09-28

**Authors:** Tong Zhao, Peifei Ying, Yahan Zhang, Hanyu Chen, Xingbin Yang

**Affiliations:** 1 Shaanxi Engineering Laboratory for Food Green Processing and Safety Control, College of Food Engineering and Nutritional Science, Shaanxi Normal University, Xi’an 710119, China; 2 College of Food Engineering and Nutritional Science, Shaanxi Normal University, Xi’an 710119, China; 42014096ypf@snnu.edu.cn (P.Y.); zyh1031@snnu.edu.cn (Y.Z.); chy42014046@snnu.edu.cn (H.C.)

**Keywords:** peanut meal, peanut meal hydrolysates, high-value utilization, food processing, breeding, industrial fields

## Abstract

Peanut meal (PM) is a by-product of extracting oil from peanut kernels. Although peanut meal contains protein, carbohydrates, minerals, vitamins, and small amounts of polyphenols and fiber, it has long been used as a feed in the poultry and livestock industries due to its coarse texture and unpleasant taste. It is less commonly utilized in the food processing industry. In recent years, there has been an increasing amount of research conducted on the deep processing of by-products from oil crops, resulting in the high-value processing and utilization of by-products from various oil crops. These include peanut meal, which undergoes treatments such as enzymatic hydrolysis in industries like food, chemical, and aquaculture. The proteins, lipids, polyphenols, fibers, and other components present in these by-products and hydrolysates can be incorporated into products for further utilization. This review focuses on the research progress in various fields, such as the food processing, breeding, and industrial fields, regarding the high-value utilization of peanut meal and its hydrolysates. The aim is to provide valuable insights and strategies for maximizing the utilization of peanut meal resources.

## 1. Introduction

### 1.1. Overview of Peanut Resources

Peanuts, also known as groundnuts, longevity fruit, or chitose, scientifically referred to as *Arachis hypogaea*, belong to the leguminous crop family *Panicum miliaceum* L. Peanuts, alongside rapeseed, soybean, sunflower seed, and cottonseed, are one of the world’s five major oil crops with widespread cultivation and production globally. According to the data from the website of Statista, peanut oil production is estimated to be around 5.86 million metric tons from 2021 to 2022, accounting for approximately 3.14% of the world’s total annual output of edible vegetable oil [1]. Based on the FAO’s enterprise statistical database, China was the largest peanut producer in 2021, with an output of 18,307,800 tons, followed by India and Nigeria with outputs of 10,244,000 tons and 4,607,669.46 tons, respectively. In 2020, the global production of peanut oil reached 4.61 million tons [2], which sufficiently illustrates the significance of peanuts in agricultural production and trade [3].

Unlike most plants, peanut fruits grow underground rather than above the ground. A complete peanut consists of a peanut shell, a peanut seed coat (also known as the red coat), a peanut kernel, and a peanut embryo. Peanuts are rich in protein and fat, with the peanut embryo containing 26–38% protein and 42–46% oil, while the peanut kernel contains 25–36% protein and 47–53% oil [4]. In addition to protein and fat, peanuts also contain about 15% carbohydrates, mainly including starch, oligosaccharides, sucrose, raffinose, stachyose. Recent studies have found that oligosaccharides, stachyose, and raffinose can promote the proliferation of beneficial gut bacteria, providing excellent intestinal protection [5,6,7,8,9,10]. Meanwhile, peanuts are a good source of water-soluble and fat-soluble vitamins. For example, water-soluble vitamins include riboflavin, thiamine, niacin, and choline [11]. Fat soluble vitamin: vitamin E, also known as α-tocopherol, has a variety of biological functions. It is not only commonly used as an antioxidant but has also been found to be involved in the immune response and plays an important role in neurodevelopment and cognitive function according to recent studies [12,13].

Peanuts also contain other types of bioactive substances, such as polyphenols, phytosterols, triterpenes, and alkaloids [14]. Among them, the main polyphenols in peanuts are flavonoids (such as procyanidins) and phenolic acids (such as p-coumaric acid, caffeic acid, and chlorogenic acid) [11]. Polyphenols exhibit a strong antioxidant activity by scavenging free radicals through single electron transfer (SET) and hydrogen atom transfer (HAT). Meanwhile, numerous studies have demonstrated that the potent biological activity of surface polyphenols confers various benefits to human health, including the prevention of cardiovascular disease, diabetes, and obesity, as well as anti-cancer, anti-inflammatory, and antibacterial effects [15]. The phytosterols in peanuts are mainly located in the seeds and contain a variety of substances, such as β-sitosterol, rapeseed oil sterol, stigmasterol, and sitosterol [14]. As natural active ingredients in plants, phytosterols usually coexist with oils in plant seeds and pollen grains. They have structural similarities to animal sterols like cholesterol and function to reduce cholesterol levels, prevent cancer and atherosclerosis, provide anti-inflammatory effects, and act as antioxidants for human health [16]. The triterpenes found in peanuts primarily consist of five types: α-amyrine, β-amyrine, 28-methyleneobusifoliol, 24-methylenecycloartanol, and cycloartenol [17]. Gaydou et al. [18] isolated and identified a small amount of cycloartanol, butyrospermol, cycloartenol, 24-methylenecycloartanol, and cyclobranol. Peanuts contain a small number of alkaloids. Lou et al. [19] and Zwickenpflug et al. [20] isolated and identified the alkaloids in peanut samples, proving that the alkaloids present in peanuts include: (+)-α-methoxy-1H-indole-3-propanoic acid, (+)-α-hydroxy-1-methyl-1H-indole-3-propanoic acid, and 2-(3-pyridyl)-1-pyrroline.

Notably, although peanuts are rich in various nutrients and have various biological functional activities, the specific protein fractions (Ara h1 to Ara h9) contained in peanuts have been identified as allergens, capable of eliciting acute reactions even in trace amounts (~200 μg). Therefore, peanuts are one of the most common food allergies among children in western countries, and allergies caused by consuming peanuts are known as peanut allergy (PA). The prevalence rate of PA in western countries is between 1% and 2%, and some data (but not all) suggest that the incidence and prevalence rates of PA may be increasing, i.e., Sicherer et al. [21], Licari et al. [22], and Jay A. Lieberman [23]. Additionally, about 75–80% of children with PA will continue to experience it into adulthood, even throughout their lives [23]. Therefore, individuals with PA need to avoid nuts altogether to prevent peanut-induced allergic reactions such as rhinitis, dermatitis, asthma, and cross reactions with other food allergies.

Additionally, peanuts need to be fully sun-dried before storage to ensure that the moisture content of the peanut fruit is less than 8% [24]; meanwhile, they must be maintained in a ventilated and dry storage environment [25]. Inappropriate storage conditions, such as high temperature and relative humidity, can lead to the growth of *Aspergillus flavus* and *Aspergillus parasiticus* on peanuts. These fungi produce aflatoxin, which contaminates peanuts and causes adverse reactions including vomiting, diarrhea, nausea, and even more serious health issue after consumption. This significantly affects the quality and food safety of peanuts [26,27]. *Aspergillus flavus* contamination in peanuts is the primary factor that causes quality deterioration. Unfortunately, the structure and properties of aflatoxin are stable and not easily damaged by high-temperature cooking [28]. Therefore, strict control should be exercised over the storage conditions of peanuts and peanut by-products for food processing purposes, as well as their application in the food and aquaculture industry, to inhibit the growth of *Aspergillus flavus*, *Aspergillus parasiticus,* and other fungi.

### 1.2. Overview of Peanut Meal Resources

Peanut meal, also known as peanut oilcake, is a significant by-product of the peanut oil extraction process, with nearly 8 million tons being produced worldwide every year [29]. Peanut meal contains 47–55% protein, making it an excellent source of plant-based protein. Its protein content is comparable to that of soybean protein and significantly higher than that of lentils and mung beans (defatted peanut meal contains 52.2 g of protein per 100 g of raw material, while defatted soy contains 51.5 g, lentils contain 24.6 g, and green peas contain 5.4 g) [30]. Peanut meal is also an abundant source of essential amino acids [31].

Similar to peanuts, peanut meal protein still contains allergenic protein fractions (Ara h1 to Ara h9), and it is prone to being used as a substrate by *Aspergillus flavus* and *Aspergillus parasiticus* during storage and transportation. This can result in the production of aflatoxin, which seriously affects food safety and quality, while also limiting the utilization of peanut meal [32]. Currently, various methods have been employed to reduce the allergenicity of peanut meal, including heat treatment, high-pressure treatments, irradiation, acid treatment, fermentation enzymatic treatments (such as peroxidase), phytic acid, or activated charcoal. However, these methods have limited effectiveness and the heat treatment did not demonstrate any inhibition of the sensitization potential of specific protein fractions. Regarding the removal/purification of aflatoxin decontamination, it can be achieved through the use of gamma radiation (using a 60 Co source) or the ozone technique for treatment. Additionally, certain organic solvents such as hexane, ethyl acetate, and petroleum ether, as well as strong bleaching chemicals like calcium hydroxide and sodium hypochlorite, have also been proven to be effective [27]. Unfortunately, due to the high cost of processing for aflatoxin decontamination and reducing allergenicity in peanut meal, it has primarily been used as a feed for livestock or plant fertilizer, resulting in the underutilization of its abundant nutrients, including proteins, polypeptides, amino acids, fats (lipids), carbohydrate, minerals, and vitamins [33]. Therefore, it is of great significance to explore the efficient strategies for maximizing the economic value of peanut meal through high-value utilization.

### 1.3. Overview of Peanut Meal Hydrolysates

Although peanut meal is rich in protein resources, most of them are not suitable for direct consumption. Mild acid, alkali, and enzyme hydrolysis can be used to obtain small molecule polypeptides that are more easily absorbed and utilized. However, the denaturation of peanut protein occurs during acid or alkali hydrolysis, leading to the production of insoluble large protein subunits (30 kDa–70 kDa), which hinders the development of a broader range of peanut meal in food processing [32].

Therefore, adopting enzyme hydrolysis has been proven to produce amino acids that are easily and quickly absorbed compared to intact protein. Additionally, hydrolyzed peanut protein is more effective in improving functional properties. Generally, there are several enzymes used for protein hydrolysis, such as Alcalase, Neutrase, Protizyme, Flavourzyme, neutral proteases, alkaline endopeptidases, pancreatin, and papain [32,34]. Zhao et al. [35] used a commercial protease to hydrolyze hot-pressed peanut meal by enzyme and extract protein, resulting in an increased protein utilization rate of 60.61–67.86%. The DPPH free radical scavenging ability of the enzymatic hydrolysis products from hot-pressed peanut meal was higher than that of low-pressed peanut meal. Yu et al. [36] also discovered that the highly denatured peanut protein is more easily hydrolyzed by proteases compared to undenatured peanut protein treated at a low temperature, offering a mild, environmentally friendly, and energy-saving enzymatic hydrolysis process. Furthermore, the resulting hydrolyzed product is of a high quality and holds broad prospects for application. Therefore, enzymatic hydrolysis technology has become a suitable method for the further valuable utilization of hot-pressed peanut meal.

## 2. Classification and Characteristics of Peanut Meal

**Figure 1:**
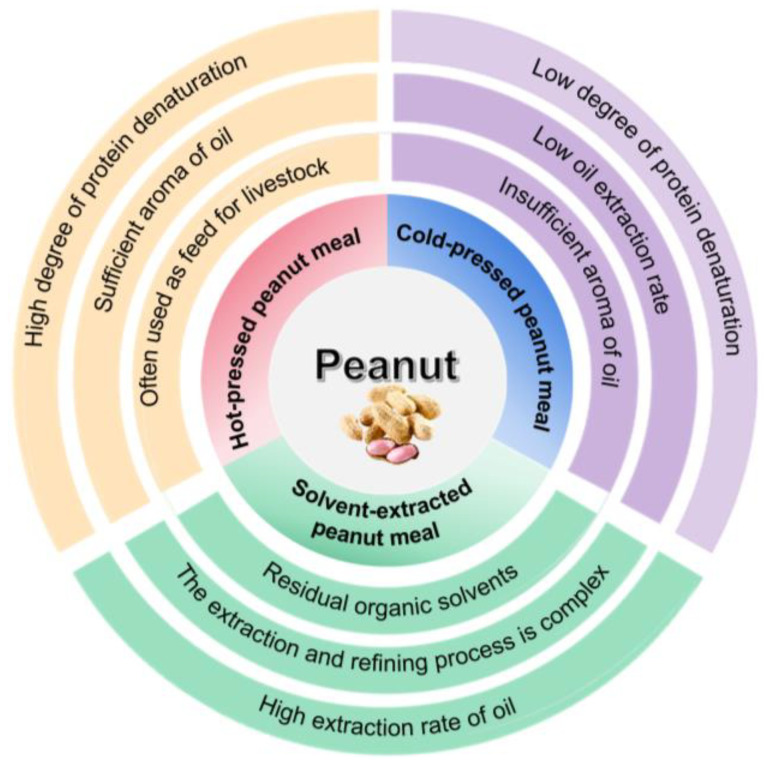
The classification and features of three types of peanut meal.

Peanut meal is a by-product that is generated during the process of extracting oil from shelled peanuts through low-temperature secondary pressing or high-temperature pressing. Typically, peanut meal appears as small granules or a powder with a light peanut flavor, containing a few peanut shells and having a red color or brown color. In terms of texture, the peanut meal exhibits an excellent uniformity and fluidity [37]. Depending on the different processes used for peanut oil extraction, peanut meal can be classified into three types: hot-pressed peanut meal, cold-pressed peanut meal, and solvent-extracted peanut meal. The classification and features of three types of peanut meal are depicted in Figure 1.

Hot-pressed peanut meal is a by-product derived from peanuts that have undergone high temperature pressing (exceed 120 °C) [38]. The heat generated during this process induces the Maillard reaction between proteins and polysaccharide in peanuts, resulting in significant protein denaturation under high-temperature treatment and a subsequent reduction in its nutritional value and functional properties. Consequently, hot-pressed peanut meal is commonly utilized as a feed for livestock [39]. However, it is worth noting that hot-pressed peanut meal exhibits antioxidant activity attributed to the presence of melanoidins. Moreover, in comparison with the hydrolysate of cold-pressed peanut meal, the hydrolysate of hot-pressed peanut meal demonstrates a higher concentration of small peptides and free amino acids, indicating a more potent antioxidant activity [39,40,41].

The production of cold-pressed peanut meal occurs through the process of pressing peanut oil below 60 °C, which effectively prevents the denaturation of peanut protein due to the low-temperature treatment. As a result, the nutritional content in the peanut meal is better preserved. Typically, cold-pressed peanut meal contains approximately 47–55% protein content with a well-balanced ratio of amino acids, making it an excellent source of edible protein. However, it is important to note that the yield of cold-pressed peanut meal is relatively low and specialized equipment is required to maintain the low-temperature conditions. These factors contribute to the higher cost and more stringent requirements associated with the production of cold-pressed peanut meal [39,42].

Solvent extraction is a method that involves the use of organic solvents, such as n-hexane or ethyl acetate, for the immersion of peanuts. These solvents chemically react with the oil present in peanuts, facilitating the separation of oil from the peanuts and enabling peanut oil production. The by-product obtained from this oil extraction process is referred to as solvent-extracted peanut meal [43]. The yield of peanut meal obtained through solution extraction is significantly high, and the resulting peanut meal often retains a delicate aroma while providing good protection for the peanut meal protein.

## 3. The Nutrient Composition of Peanut Meal

**Figure 2:**
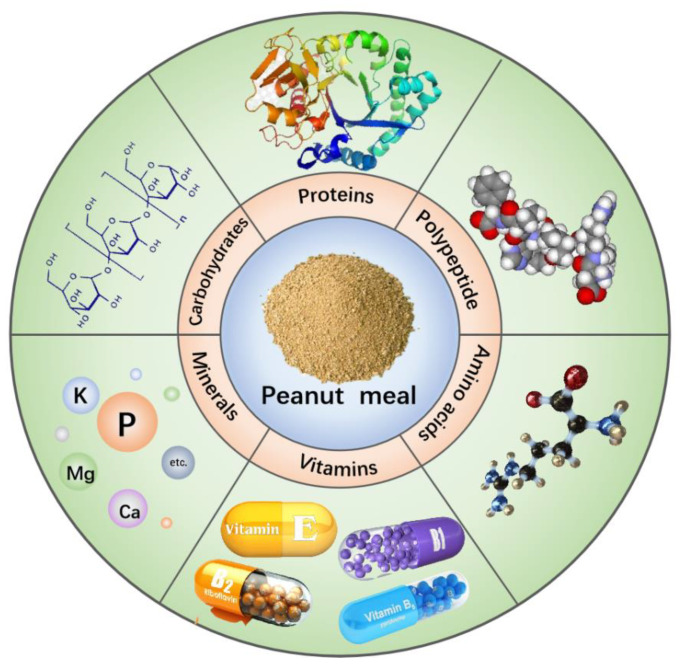
The nutritional composition of peanut meal.

Peanut meal, a by-product of peanut pressing, is abundant in protein, polysaccharides, minerals, vitamins, and other nutrients. The nutritional composition of peanut meal is illustrated in Figure 2.

### 3.1. The Protein in Peanut Meal

Protein is always important for human beings. In comparison with animal protein, although animal proteins are the primary source of balanced amino acids and are more easily absorbed by human beings, there has been an increasing demand for plant protein in recent years due to its low cost and potential as an alternative to animal protein in food processing [44]. As a plant-based option, peanut meal contains abundant plant protein, with a total protein content of around 48.68% [29,45]. It includes 10% water-soluble proteins, which usually have a small molecular weight and can easily dissolve in water, as well as about 90% salt-soluble proteins such as conarachins [46]. Salt-soluble proteins have a high molecular weight but can dissolve in saltwater due to the presence of salt. These salt proteins play a role in maintaining quality during food processing by encapsulating water through intense intermolecular movement when exposed to heat and forming stable structures under the complexation of certain ions, thereby achieving stable quality.

Due to the high nutritional value and low cost of peanut protein, it has been proven to offer multiple potential health benefits [11,47,48]. Compared to soybean protein, the protein found in peanut meal has a lower level of anti-nutritional factors. This makes it easier for the human body to digest and absorb. Additionally, the high solubility index of nitrogen in peanut protein makes it suitable for incorporation into various food products. Peanut meal protein can be used to produce peanut protein beverages as a substitute for animal milk protein or as a protein source in food products for individuals with lactose intolerance, making it more inclusive for a wider range of consumers. Until now, a great number of nutritional drinks containing peanut protein have already been developed [49,50,51,52]. Moreover, peanut protein concentrates have been used in sausages, bologna, pasta sauce, and salad dressings due to their exceptional water-holding and emulsifying capacity which provide unique taste and flavor characteristics [53,54]. Table 1 compares the functional properties of peanut meal protein with those of other commonly seen plant proteins.

### 3.2. Peanut Polypeptide in Peanut Meal

The special peptide sequences remaining after peanut protein hydrolysis are called peanut polypeptides, which consist of 3–6 amino acids. Due to their outstanding biological activities such as antioxidant, anti-inflammatory, and antibacterial activities, low sensitization rate, and easy digestion and absorption in the body, peanut peptides have been widely used in the development of nutritional products such as lipid-lowering, blood-pressure-lowering, and antibacterial products and those used in fatigue reduction and immunity enhancement [68]. Additionally, peanut polypeptides possess physicochemical properties such as a good water solubility, emulsification, foaming capacity, thickening capacity, and plasticity. Therefore, they can be applied in various industries such as food processing, healthy product production, pharmaceutical manufacturing, aquaculture, and chemical engineering [69].

### 3.3. Amino Acids in Peanut Meal

The protein of peanut meal is a complete protein, with an amino acid content up to 52.2 g/100 g [32]. The amino acid profile of peanut encompasses all 20 essential and non-essential amino acids, with peanut meal being recognized as one of the most abundant sources of arginine, constituting 12.5% of the total protein content. However, the levels of methionine (0.52 ± 0.04%) and cysteine (0.64 ± 0.07%) in peanut meal are relatively low [33,70]. Fortunately, the digestibility and absorption efficiency of peanut amino acids in the human body is approximately 90%, which underscores their pivotal role in maintaining human health and promoting child growth [71].

### 3.4. Carbohydrates in Peanut Meal

In addition to peanut protein, carbohydrates are another important nutritional component found in peanut meal. Peanut meal comprises approximately 38% total carbohydrates, primarily consisting of starch (12.5%) and dietary fibers (15.8%). The dietary fibers consist of insoluble polysaccharides (cellulose and hemicellulose) and soluble oligosaccharides (raffinose, stachyose, and verbascose). Typically, the fiber content of dry peanut meal is 8.4 g/100 g, while the total defatted peanut meal is 15.8%, which is comparable to that of defatted soybean meal (17.5%) [11]. Soluble fibers possess potent scavenging abilities against hydroxyl radicals, oxygen radicals, and DPPH radicals in vivo. This characteristic enables peanut polysaccharides to exhibit potential antioxidant properties, as well as benefits such as liver protection, immune system enhancement, blood cholesterol level reduction, improved bowel movement, maintenance of a healthy weight, and other bio-functional activities [72]. Recent research has reported that a higher intake of dietary fiber is associated with a reduced risk of developing metabolic syndrome [73].

### 3.5. Fats (Lipids) in Peanut Meal

As one of the major oil crops in the world, peanuts exhibit a high content of fats (lipids) and fatty acids. The majority of these fatty acids are healthy unsaturated fats, including monounsaturated and polyunsaturated fats (Table 2), such as omega-6 and omega-3 fatty acids [74]. Monounsaturated fats can promote arterial clearance, thereby facilitating the maintenance of healthy blood flow and reducing the risk of atherosclerosis, heart attack, or stroke [11]. The extent of oil extraction during the pressing process typically serves as the primary determinant influencing the fat content of peanut meal. Higher levels of fats and fatty acids can be obtained if less oil is extracted during processing. However, it should be noted that peanut and peanut meal are susceptible to lipid autoxidation due to their high fat (lipid) and fatty acid contents. This oxidation primarily occurs during storage and is influenced by factors such as humidity, temperature, oxygen exposure, and light [25].

### 3.6. Minerals in Peanut Meal

Peanut meal is abundant in essential mineral elements, including magnesium (Mg), potassium (K), calcium (Ca), iron (Fe), sodium (Na), and zinc (Zn) as indicated in Table 3. Among these minerals, Mg, K, Ca, and Fe plays pivotal roles in maintaining human physiological functions. Consequently, peanut meal can serve as a valuable source of mineral nutrition for the general population [33].

### 3.7. Vitamins in Peanut Meal

Apart from macronutrients, peanut meal also contains vitamins, with vitamin B and vitamin E being the two main vitamins found in peanut meal. The vitamin B group consists of water-soluble vitamins, including vitamins B1–B17 as well as vitamin B-h, vitamin B-w, and vitamin B-x. Among the vitamin B group, peanut meal mainly contains vitamin B1, vitamin B2, and vitamin B6 with contents of 4.22 mg, 1.65 mg, and 5.44 mg per 1000 g of peanut meal, respectively, according to Chen’s study [75]. Vitamin B1, also known as thiamine, possesses the ability to suppress cholinesterase activity while enhancing skin health through its anti-inflammation properties that mitigate seborrheic dermatitis and eczema. Vitamin B2 or riboflavin, plays a crucial role in cellar redox reactions along with facilitating hemoglobin synthesis and metabolizing carbohydrates, protein, and fat. Moreover, it aids in sunburn protection while stimulating cellular rejuvenation. An insufficient intake of vitamin B2 renders the skin susceptible to sunlight-induced sensitivity resulting in facial erythema accompanied by pruritus or potentially leading to solar dermatitis. Vitamin B6 exhibits a profound impact on maintaining optimal dermal well-being due to its capacity for diminishing blood capillary permeability and hyaluronidase inhibition whilst concurrently fostering amino acid metabolism. Furthermore, it effectively suppresses allergic reactions and inflammatory responses that trigger skin allergies while promoting the growth of epithelial cells to maintain skin health [76,77]. In terms of vitamin E, also known as tocopherol, peanut meal contains 0.67 mg of vitamin E per 1000 g [75]. As a crucial fat-soluble vitamin, vitamin E possesses various bioactivities, particularly in maintaining the connective tissue elasticity, protecting skin mucosa, improving microcirculation in hair follicles, and enhancing blood circulation.

## 4. The Applications of Peanut Meal and Its Hydrolysates

As one of the most significant oil-producing crops worldwide, peanuts generate a multitude of by-products during the oil extraction process. Despite containing various nutrients such as protein, carbohydrates, and polyphenols, these peanut by-products in peanut meal have been primarily used in livestock feed for a long time with limited high-value applications. Fortunately, recent decades have witnessed an increasing focus on exploring the high-value utilization of peanut meal through methods like modification, (enzymatic) hydrolysis, and crosslinking, among others. Consequently, the by-products derived from peanut oil extraction possess versatile applications across different fields.

### 4.1. The Application of Peanut Meal and Its Hydrolysates in the Field of Food Processing

In order to enhance the utilization of peanut meal in the food industry, enzymatic hydrolysis and fermentation methods are commonly employed for texture, structure, and flavor modification. These treatments facilitate the conversion of peanut meal samples into food flavor agents (Table 4). For instance, Zhang et al. [78] used protease to hydrolyze protein from nonfat peanut meal and evaluated the physicochemical properties and sensory attributes of the resulting products. Various enzymes such as GA (Genencor Alkaline Protease), GN (Genencor Neutral Protease), and Protamex were used for hydrolyzing nonfat peanut meal protein. By assessing both the physicochemical properties and sensory attributes of the hydrolyzed products, it was observed that GA- and Protamex-treated products exhibited astringent and bitter taste profiles while GN-treated products possessed a pleasant umami flavor. This underscores their potential as food flavoring agents following enzymatic-hydrolysis-induced modifications in sensory properties. In terms of fermentation treatment, Zeng et al. [79] developed a bacterial fermentation process using *Bacillus subtilis* on peanut meal based on enzymatic hydrolysates to generate high-value-added products with desired flavors and an elevated nutritional content. Furthermore, the Maillard reaction enhanced both the flavor profile and antioxidant activity of peanut meal’s hydrolysates, rendering them suitable condiments capable of enhancing both the taste experience and beneficial antioxidant properties in various foods. Wang et al. [80] used a flavor protease and trypsin to hydrolyze peanut meal. Through the electronic tongue test, the results revealed that hydrolysates exhibited a strong umami taste followed by saltiness then sourness, indicating its potential candidacy as a food condiment.

Additionally, peanut meal can undergo further processing to yield plant-based protein meat. Zhang et al. [81] utilized a high-water extrusion method through an extruder to process peanut meal, resulting in the modification and unfolding of the molecular structure of peanut protein. Subsequently, through the influence of hydrogen bonds and disulfide bonds, the molecules were reorganized and gradually formed a fibrous meat-like structure, ultimately creating a form of “plant-based protein meat”.

Due to its high protein content of superior quality, peanut meal can be enzymatically hydrolyzed using Alcalase and other enzymes to extract bioactive peptides for the development of nutritious functional foods. The resulting peptides exhibit diverse tastes and bioactivities depending on the extent of hydrolysis [82]. Ye et al. [83] utilized Alcalase to hydrolyze peanut meal and obtain a mixture of peptides, which were subsequently combined with selenium nanoparticles (SeNPs) through electrostatic interaction, leading to the formation of composite colloidal particles comprising peanut meal peptides and selenium. These particles were further utilized in the development of nutritional functional food products. Zhang et al. [85] employed papain-hydrolyzed peptides and microwave extraction techniques to break down peanut meal into small-molecule peptides that demonstrated multiple bioactivities, including antioxidant properties, free radical scavenging abilities, and angiotensin converting enzyme (ACE) inhibition. Consequently, these papain-hydrolyzed peanut peptides could be applied as ingredients in functional foods. In a different approach, Kahlon et al. [86] did not subject peanut meal to hydrolysis but instead used it as a primary raw material ingredient along with okra and sorghum for the production of a snack called sorghum–peanut meal–okra.

In recent decades, a growing number of studies have focused on the fermentation of peanut meal by probiotics in order to enhance its economic value and unlock its multifunctional properties. For instance, Jiang et al. [87] conducted a fermentation process using *Bacillus subtilis* on peanut meal and observed that the fermented peanut meal improved the learning and memory abilities of mice with dysbiosis. Additionally, it helped to alleviate intestinal disorders without any adverse effects on mice growth. Similarly, Zhang et al. [68] also utilized *Bacillus subtilis* for fermenting peanut meal, resulting in the production of hydrolyzed peanut peptides. Interestingly, they found that a longer fermentation time led to an increased content of peanut peptides; moreover, highly hydrolyzed peanut peptide exhibited an enhanced antioxidant activity and higher levels of acidic amino acids. These studies demonstrate the potential of probiotic fermentation to enhance the nutritional and functional properties of peanut meal, leading to the development of value-added products with improved health benefits.

### 4.2. The Application of Peanut Meal and Its Hydrolysates in the Field of Breeding

**Figure 3:**
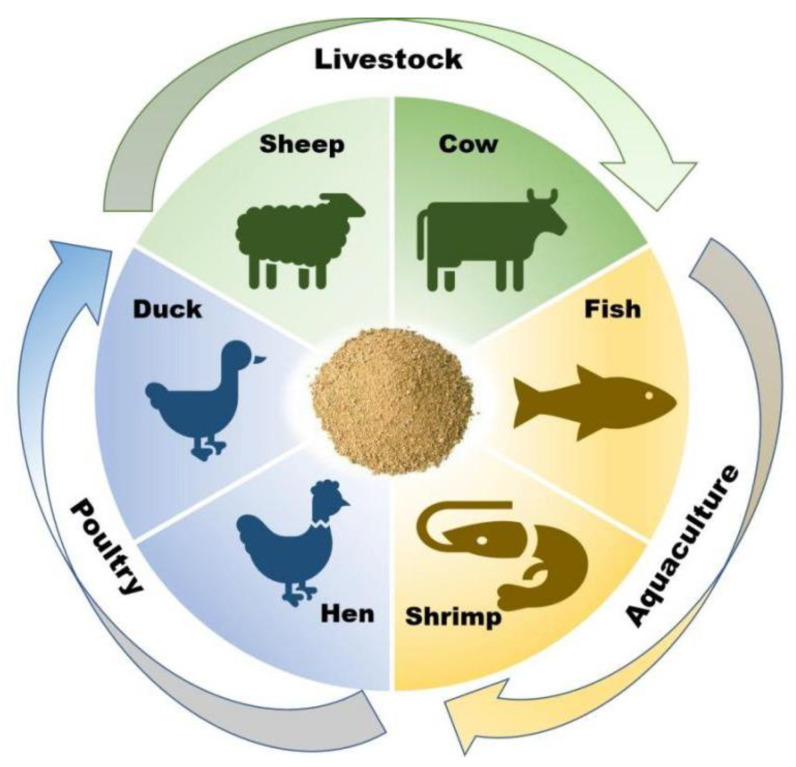
The applications of peanut meal and its hydrolysates in the field of breeding.

The by-products generated from oil extraction, namely peanut meal and soybean meal, exhibit similarities in terms of the abundant protein and dietary fiber contained. Typically, soybean meal surpasses peanut meal in terms of its higher vitamin and amino acid content. However, due to the coarser flavor profile of these by-products, peanut meal and soybean meal have primarily been utilized in livestock and poultry breeding for an extended period. It is worth noting that compared to soybean meal, peanut meal contains a higher oil content and is more susceptible to bacterial contamination. As a result, peanut meal is frequently employed in the field of livestock breeding as a partial substitute for the original feed (such as soybean meal). Conversely, in aquaculture, peanut meal is used partially as a replacement for fish meal (Figure 3, Table 5).

#### 4.2.1. The Application of Peanut Meal and Its Hydrolysates in the Aquaculture Field

Fish meal is consistently in high demand within the aquaculture industry. However, fish meal resources are constantly influenced by various factors, such as climate variability (e.g., El Niño events) and price fluctuations [88,102,103,104]. Consequently, an increasing number of individuals have opted to produce fish meal using plant-based proteins [105,106]. Amongst a plethora of plant proteins available, soybean meal and peanut meal have been identified as exceptional raw materials for fish meal production due to their elevated levels of crude protein and essential amino acids.

Ye et al. [88] investigated the substitution of fish meal with peanut meal as a feed for the juvenile hybrid grouper (*Epinephelus fuscoguttatus* ♀ × *Epinephelus lanceolatus* ♂) and assessed its effects on growth performance, immunity, and gut microbiota over a duration of 10 weeks. The findings revealed that the replacement of fish meal with peanut meal did not yield any significant effects on growth performance, feed utilization, somatic indices, or whole-body composition (*p* > 0.05). Li et al. [107] used peanut meal as a substitute for the original cottonseed protein or soybean protein in the diets of Channel Catfish. Following a 9-week investigation, the findings demonstrated that peanut meal can effectively replace up to 25 wt% of the original cottonseed meal or soybean meal without any detrimental impact on fish growth, feed efficiency, and the body composition of fish.

Vo et al. [90] assessed the suitability of three types of processed peanut meals, namely untreated peanut meal (UPM), fermented peanut meal (FPM), and germinated peanut meal (GPM), as potential replacements for fish meal in barramundi (*Lates calcarifer*) diets within a commercial aquaculture setting. The findings revealed that these peanut meals could partially substitute fish meal in the diet of juvenile barramundi, with a 60% replacement of GPM and UPM resulting in increased lipid droplet accumulation in the liver, reduced myodegeneration in muscle tissue, and reduced acidic mucin content in the distal gut.

#### 4.2.2. The Application of Peanut Meal and Its Hydrolysates in the Poultry Breeding Field

In the field of poultry breeding, peanut meal can serve as a viable alternative to soybean meal in terms of protein and unsaturated fatty acid content, thereby effectively balancing the amino acid profile and enhancing unsaturated fatty acid levels [101]. Xia et al. [96] conducted a study to assess the impact of replacing soybean meal with peanut meal on egg production, egg quality, oxidative status, and yolk fatty acid profile in laying ducks. The findings revealed that up to 75% substitution of soybean meal with peanut meal had no detrimental effects on the egg-laying performance or egg quality in laying ducks. However, the complete replacement of soybean meals by peanut meal resulted in a reduced egg production and antioxidant capacity of eggs, along with an elevated ω-6 and ω-3 fatty acid ratio in the yolk. Saleh et al. [95] incorporated peanut and linseed meal into broiler feed along with an enzyme mixture comprising xylanase, cellulase, β-mannanase, phytase, α-amylase, and protease. Following a 35-day feeding period, they found that the addition of exogenous protease and 50% peanut meal enhanced the peptide and amino acid absorption efficiency while also upregulating mRNA expression in the duodenum. Pesti et al. [98] compared two types of diets at three protein levels (16, 18.5, and 21%) for 22-to-34-week-old commercial Leghorns, where one group was a corn–peanut meal-based diet and the other group was a corn–soybean meal-based diet. The results demonstrated that during the initial 6-week period, hens fed with peanut meal exhibited a slight reduction in egg size (*p* < 0.05), while no significant difference in egg size was observed during the latter 6 weeks (*p* > 0.14). In terms of egg quality, hens fed with peanut meal demonstrated a superior interior quality at both 26 and 30 weeks of age. Moreover, following a 2-week storage experiment, eggs from hens fed with peanut meal displayed higher Haugh units when refrigerated (4 °C, *p* < 0.05) or stored at room temperature (20 °C, *p* < 0.10) compared to those from soybean meal-fed hens. Additionally, the specific gravity of eggs from hens fed with peanut meal was marginally lower than that of soybean meal-fed hens. These finding suggests that incorporating peanut meal into laying hen diets can be advantageous.

#### 4.2.3. The Application of Peanut Meal and Its Hydrolysates in the Livestock 

##### Breeding Field

Peanut meal has been a primary component of livestock feed for an extended period, including cows, sheep, pigs, and horses. For example, dos Santos et al. [99] conducted an experiment using peanut meal to replace soybean meal in the diet of 12 lactating cows for 60 days. The results exhibited that substituting soybean meal with peanut meal had no adverse effect on the dry matter and nutrient intake and digestibility in cows. Moreover, there was no significant difference observed in microbial protein synthesis and nitrogen balance, the microbial nitrogen synthesis and microbial protein synthesis efficiency presented no difference either. Additionally, the nitrogen balance and retention rates were also similar. Therefore, regions where peanut meal is more cost-effective can entirely replace soybean meal in dairy cow diets without affecting nitrogen balance or microbial protein synthesis.

In terms of sheep, de Lima Valença et al. [101] conducted a study on 40 uncastrated male Ile de France lambs, wherein soybean meal was partially substituted with peanut meal to evaluated the microbiological and physicochemical characteristics of fresh and aged semimembranosus muscle in lambs. The results showed that peanut meal can be utilized as a partial replacement for soybean meal in lambs’ diets without compromising the quality of lamb meat.

### 4.3. Application of Peanut Meal and Its Hydrolysates in Industrial Field

As a by-product of oil extraction, peanut meal has limited industrial applications. However, considering its protein content, which contains numerous active groups, particularly globulin, it could serve as a valuable source for industrial processing. Currently, several plant proteins have been utilized in the industrial field, such as soybean protein, wheat, and zein, as well as some relatively lesser-known proteins such as sunflower proteins, pea proteins, and mung bean proteins. Therefore, the potential exists to apply peanut protein and its hydrolysates in the industrial field, and the applications of that are illustrated in Table 6.

#### 4.3.1. The Utilization of Peanut Meal and Its Hydrolysates for Plant Protein-Based 

##### Adhesives Preparation

The wood adhesive industry has long relied on petroleum-based chemicals for their exceptional performance and cost-effectiveness. However, these adhesives derived from petroleum have the potential to emit formaldehyde, thus giving rise to significant environmental concerns.

In recent years, plant-based adhesives have garnered significant attention due to their renewable and environmentally friendly properties. When it comes to plant-based adhesives, soybean meal, a by-product generated during the extraction of soybean oil, has long been the preferred choice for fabricating protein-based wood adhesive. However, challenges such as a low solid content, high viscosity, and limited water resistance have impeded the widespread application and development of soybean meal-based adhesives in various industries [111,118]. In order to enhance the adhesion performance of plant protein-based wood adhesives, peanut meal has been increasingly considered as a viable material for their preparation. Chen and colleagues [114] used hot-pressed and cold-pressed peanut meal as raw materials to develop two distinct types of plant-based adhesive. Through a series of tests, they found that the treatment temperature and modifier quantity were not critical factors. However, incorporating hot-pressed peanut meal and adding SDS significantly enhanced the adhesive strength of the peanut meal-based adhesive, which could achieve an average value of 1.05 ± 0.07 MPa. The utilization of peanut meal by Li et al. [115] in the preparation of wood adhesives through SDS and ethylene glycol diglycidyl ether (EGDE) modification resulted in a significant improvement (90%) in water resistance, meeting the requirements for interior wood use. This enhancement can be attributed to the disruption of the structure of peanut meal protein caused by added SDS, allowing for reaction with EDGE to form a dense network.

Peanut meal with soybean meal was blended by Li and colleagues [111] to prepare a plant-based wood adhesive using different weight ratios of materials. After characterizing the physicochemical properties, including the amino acid composition, molecular weight distribution, solid content, viscosity, hydrolytic stability, morphological properties, and functional groups, it was observed that the resulting adhesives from the blended mixture exhibited a decreased viscosity and a higher solid content compared to those made solely from soybean meal. Furthermore, when 20% peanut meal was added as an optimum ratio, the wet and dry shear strength of the plywood bonded with this adhesive increased by 50% (1.02 ± 0.02 MPa) and 31% (1.55 ± 0.03 MPa), respectively. This improvement in bonding performance can be attributed to the optimized protein structure and side chains promoting cross-linking reactions.

Chen et al. [113] also advocated for the utilization of peanut meal protein-based wood adhesives in the plywood industry. They used urea (U) and epichlorohydrin (ECH) to augment the adhesive properties, while investigating the underlying modification mechanism. The results revealed that incorporating U and ECH into peanut meal-based wood adhesives resulted in an enhanced water resistance, increased the apparent viscosity, and provided a higher solid content compared to those without U and ECH addition. This improvement can be attributed to the denaturation of peanut meal protein by U, which exposed more reactive groups, facilitating accelerated reactions with ECH-treated peanut meal protein, ultimately leading to the formation of a dense and cross-linked network. Furthermore, it is noteworthy that employing U and ECH in adhesives yielded a remarkably smooth protein surface that effectively impeded moisture penetration and improved the water-resistance capacity.

Qu et al. [112] employed sodium dodecyl sulfate (SDS) denaturation, nano-silica (nSiO_2_) reinforcement, and polyamide polyamine epichlorohydrin resin (PAE) to facilitate the hot-pressed peanut meal protein (HPMP) adhesive with enhanced bonding strength achieved through a three-step cross-linking process. It was observed that the boiling water strength of the HPMP adhesive exceeded the bonding strength requirement specified by the China National Standards for Class I plywood, measuring at 0.85 MPa compared to the required 0.7 MPa. Additionally, the HPMP adhesive exhibited an extended mildew resistance for at least 12 days while experiencing a significant increase in the solids content by 45.4% and viscosity by 274.6%.

#### 4.3.2. The Utilization of Peanut Meal and Its Hydrolysates for Biosurfactant Preparation

Biosurfactants are amphiphilic compounds produced by microorganisms such as bacteria, yeasts, and fungi under specific culture conditions. They exhibit a high activity, excellent emulsification performance, intricate spatial structure, low surface tension (typically below 30 mN/m), remarkable chemical stability, and thermal stability. Moreover, owing to their environmental friendliness and a wide availability of raw material sources, they can be extracted from industrial waste or agricultural products. As the by-products generated from peanut oil extraction, peanut meal can be enzymatically converted into a glycolipid anionic biosurfactant called rhamnolipid by *Pseudomonas aeruginosa* [108]. In comparison to chemical surfactants, rhamnolipid is more environmentally friendly and more easily degraded, exhibiting a high activity and non-toxicity. It finds applications in the field of oil exploitation for reducing the interfacial tension between oil and water, thereby enhancing the displacement efficiency of crude oil and reducing costs. Moreover, in agriculture, rhamnolipid can stimulate growth in crops, vegetables, fruits, and flowers while aiding nutrient absorption and improving the efficacy of pesticides and fertilizers. Additionally, rhamnolipids can serve as bulking agents to increase the baking volume and flavoring agents in food processing. Kane and colleagues [109] employed three different enzymes (Alcalase, Flavourzyme, and pepsin) to hydrolyze peanut meal resulting in highly antioxidant hydrolysates with enhanced emulsifying properties and foaming ability. This suggests that hydrolyzed peanut meal proteins can act as a surfactant at air/water or oil/water interfaces for foams or emulsions, respectively.

#### 4.3.3. The Utilization of Peanut Meal and Its Hydrolysates for Films Preparation

Due to its non-toxic biodegradable nature and excellent water vapor permeability, peanut meal has gained popularity in the production of films [69]. However, the inherent hydrophilicity of plant proteins often leads to a decrease in mechanical properties (such as strength and elongation) and water resistance [119], necessitating the addition of plasticizers to enhance these shortcomings. Reddy et al. [117] utilized citric acid as a cross-linking agent to form crosslinked films with peanut meal protein, which exhibited an unaffected water vapor permeability while significantly improving in dry and wet tensile strengths compared to pure peanut protein films. Another experiment involving glycerol as a plasticizer demonstrated that by incorporating glycerin into peanut meal followed by drying under high pressure and temperature conditions, the resulting fabricated peanut protein film achieved an impressive elongation of up to 63%, surpassing films made from peanuts or other commonly used plant proteins. These findings indicate the immense potential of peanut meal in thermoplastic product processing.

## 5. Conclusions

Peanut meal is abundant in protein and contains a diverse range of nutrients, making it an economical source of protein. However, its utilization has primarily been focused on animal feed for livestock and aquaculture, with only a limited portion being applied in the food industry, chemical industry, or other fields at present. This review emphasizes the application of peanut meal in the food industry, highlighting its potential to be modified through enzymatic hydrolysis and fermentation for use as a flavoring agent or additive in food products. Additionally, it can be processed into “meat substitutes” or incorporated into functional foods to enhance their nutritional value. Thus far, despite the numerous advancements and applications of peanut meal, effective measures must still be taken to prevent microbial contamination, such as *Aspergillus* L. during storage and processing (e.g., hydrolysis and fermentation), in order to ensure the biosafety and processability of peanut meal and its processed products. In the future, there is a need to explore the high-value utilization of peanut meal across multiple fields. Employing more scientific and technological methods can help to extend the shelf life of peanut meal and its hydrolysate, enhance their nutritional value in aquaculture, and improve their functional characteristics in industry [120], thus achieving high-value utilization across various domains.

## Figures and Tables

**Table 1:** The comparison of functional properties between peanut meal protein with those of other commonly seen plant proteins.

Raw Material	Emulsification Activity Index (m^2^/g)	Emulsion Stability (min)	Foaming Capacity (%)	Foam Stability (%)	Water-Holding Capacity (g/g)	Oil-Holding Capacity (g/g)	References
Defatted peanut meal (PM)	42.5 ± 4	18.5 ± 3	60 ± 6	50.2 ± 1.2	0.9 ± 0.07	0.9 ± 0.1	[54]
Peanut protein concentrate	50.3 ± 2.1	36.1 ± 7.8	53.1 ± 0.9	40.2 ± 2.2	0.8 ± 0.1	2.01 ± 0.6	[55]
Peanut protein isolate	60.5 ± 2	22.3 ± 5	58 ± 7	25 ± 5	0.2 ± 0.05	1.6 ± 0.02	[54]
Bean protein isolate	15.6 ± 0.6 to 22.0 ± 0.7	60.1 ± 1.5 to 164.2 ± 12.9	72 ± 2 to 91 ± 1	71 ± 1 to 82 ± 5 (30 min)	1.8 ± 0.1 to 2.1 ± 0.1	4.0 ± 0.3 to 5.4 ± 0.3	[56]
Soybean protein isolate	Around 17 to 32	10.5 to 11.5	Around 180	Around 178	4.07	3.60	[57,58,59,60]
Pea protein isolate	42.87 ± 0.80	12.40 ± 0.04	145.6	58.0	0.3 to 2.6	5.3	[61,62,63]
Chickpea protein isolate	47.90 ± 1.88	82.94 ± 3.18	113.1	60	2.3 to 2.9	2.1 to 4.0	[62,63,64]
Lentil protein isolate	44.51 ± 1.06	86.79 ± 4.14	425% at pH 3; 403% at pH 5; 410% at pH 7	79% at pH 3; 82% at pH 5; 84% at pH 7	1.04	8.62	[63,65,66]
Mung bean protein	63.18 ± 0.38	62.75 ± 0.43	89.66	78.33 ± 0.57 (30 min)	3.18	3.03	[60]
Red kidney bean protein	24.2 ± 2.9	68.3 ± 7.1	76.7 ± 5.6	54.5 ± 0.71 (60 min)	2.07 ± 0.001	2.39 ± 0.04	[67]

**Table 2:** Fatty acids in peanuts [11].

Fatty Acids	Numerical Abbreviation	Fatty Acids	Numerical Abbreviation
Palmitic	C16:0	Arachidic	C20:0
Palmitoleic	C16:1	Paullinic	C20:1
Stearic	C18:0	Docosanoic	C22:0
Oleic (n-9)	C18:1	Lignoceric	C24:0
Linoleic (n-6)	C18:2		
Linoleic (n-3)	C18:3		

**Table 3:** Mineral contents in peanut meal [33].

Mineral Elements	Content (%)	Mineral Elements	Content (ppm)
P	0.57 ± 0.06	Na	117 ± 54
K	1.22 ± 0.12	Fe	542 ± 465.4
Ca	0.08 ± 0.02	Al	423 ± 348
Mg	0.31 ± 0.04	Cu	12 ± 2
		Zn	56 ± 6
		Mn	33 ± 5

**Table 4:** The applications of peanut meal and its hydrolysates in the field of the food processing industry.

Products	Applications	Methods	References
Peanut meal (PM) hydrolysates	Flavoring agents	Protease hydrolysis	[78]
	Flavoring agents, nutritional supplements	Protease hydrolysis Fermented with *Bacillus subtilis* Maillard reaction	[79]
Meat substitutes	Meat-like fibrous structure	High-moisture extrusion	[81]
Polypeptide	Flavoring agents	Hydrolysis of calcitase	[82]
PM peptide mixture-selenium composite colloidal particles	Play a role in nutraceutical	Hydrolysis of calcitase	[83]
Pork ham sausages	Partly substituted pork	Partly incorporated with defatted PM	[84]
Small molecule peptides	Act as functional food additive	Peptide hydrolysis by papain Microwave extraction	[85]
Sorghum–PM–okra snack	Snack	Cook	[86]
Fermented PM	Functional food	*Bacillus subtilis* fermentation	[42,78]

**Table 5:** The applications of peanut meal and its hydrolysates in the field of breeding.

Breeding Fields	Application	Materials	Treatment	Conclusions/Features	References
Aquacultural	Aquacultural (*Epinephelus*) feeding	Peanut meal (PM), fish meal (FM)	Replaced FM with PM, fed for 10 weeks	Replacing 50% FM with PM had no effect on growth, but changed the immunity and intestinal microbiota of juvenile hybrid grouper	[88]
	Aquacultural (Yellow River *Cyprinus carpio* var) feeding	Soybean meal (SM), PM, blend rapeseed plant protein (BP, contained PM, rapeseed meal, cottonseed meal)	Replaced SM by BP, fed for 60 days, 3 times a day	Replacing SM with 600 g/kg BP had no effect on the growth of Yellow River carp, but replacing SM with 800 g/kg depressed the growth of Yellow River carp	[89]
	Aquacultural (juvenile barramundi, *Lates calcarifer*) feeding	FM, untreated peanut meal (UPM), fermented peanut meal (FPM), germinated peanut meal (GPM)	Replaced FM with UPM, FPM, and GPM, respectively, fed for 8 weeks	Feeding 60% GPM and UPM increased the amount of lipid droplets in liver, increased myodegeneration in fish muscle and decreased the acidic mucins in the distal gut, slowed down the growth, and decreased the survival percentage	[90]
	Aquacultural (Channel Catfish) feeding	PM, FM	Replaced FM with PM, fed for 9 weeks	Replacing FM with 25% PM had no effect on the growth, feed efficiency, and body composition of Channel Catfish	[91]
	Aquacultural (Mozambique Tilapia Fries *Oreochromis mossambicus*) feeding	PM, FM	Replaced FM with PM, fed for 45 days	Replacing FM with 20% PM had no effect on the growth, body composition, and general health of Mozambique Tilapia Fries	[92]
	Aquacultural feeding (Pacific white shrimp, *Litopenaeus vannamei*)	PM, FM	Replaced FM with PM, fed for 6 weeks, 3 times a day	Decreased the whole-body protein and ash content of shrimp if the content of PM was above 280 g/kg	[93]
	Aquacultural feeding (Juvenile white shrimp *Litopenaeus vannamei*, *Boone*)	SM, PM, FM, lysine, methionine	Replaced FM with SM and PM, fed for 8 weeks	The addition of PM and SM decreased the plasma total cholesterol level of shrimp and decreased the digestibility of dry matter, protein, and energy contained in diets	[94]
Poultry breeding	Poultry (broilers) feeding	PM, linseed meal, enzyme mixture (contained xylanase, cellulase, phytase, β-mannanase, α-amylase, protease)	Fed for 35 days	The supplementation of protease and 50% PM improved the efficiency of peptide and amino acids absorption in broilers	[95]
	Poultry (ducks) feeding	PM, SM	Replaced SM with PM, fed for 16 weeks	Egg production improved with the increase in PM content, while egg weight and feed consumption decreased; the feed conversion ratio and egg mass both decreased when the PM content was 100%	[96]
	Poultry (broilers) feeding	Corn–PM-based diet, corn–SM-based diet, threonine (Thr)	Added amino acids to corn–PM-based diet and corn–SM-based diet, fed for 42 days	PM could be used as a protein source for broilers	[97]
	Poultry (Leghorns) feeding	Corn, PM	Fed for 30 weeks	PM-fed hens laid eggs with better interior quality at 26 and 30 weeks of age	[98]
Livestock breeding	Livestock (cows) feeding	PM, SM	Replaced SM with PM, fed for 60 days	Partly replacing SM with PM had no effect on the intake and digestibility of dry matter and nutrients of cows	[99]
	Livestock (pigs) feeding	Rice bran, corn germ meal, sunflower meal, corn gluten feed, PM	Housed in metabolism crates individually for 48 days	Pigs fed with PM and full-fat rice bran obtained the highest net energy	[100]
	Livestock (lambs) feeding	PM, SM	Replaced SM with PM, fed for 60 days	Partly replacing SM with PM in lambs diet had no adverse effect on mutton quality	[101]
	Livestock feeding	PM and *Bacillus licheniformis*	Solid-state fermented by *Bacillus licheniformis*	Improved the nutritional and antioxidant properties of PM and improved the hydrolysis of allergic proteins, digestion, and absorption of protein	[101]

**Table 6:** The utilization of peanut meal and its hydrolysates in industrial field.

Industrial Fields	Application	Materials	Treatment	Conclusions/Features	References
Surfactant	Biosurfactant	PM, *Pseudomonas aeruginosa*	Used *Pseudomonas aeruginosa* to convert PM to a glycolipid anionic biosurfactant rhamnolipid	The produced rhamnolipid reduced water surface tension and inhibited the growth of tested bacteria and fungi	[108]
	Surfactant	PM, Alcalase, pepsin, Flavourzyme	Used Alcalase, Flavourzyme, and pepsin to hydrolyze PM	Enhanced the emulsifying and foaming ability of hydrolyzed PM	[109]
Adhesives	Plant protein-based adhesives	Hot-pressed PM protein (HPMP), NaOH solution, acetic acid	Washed peanut shell with alkaline and acid, then acidified followed by reactions with ethylene glycol and ethylene glycol in turn to construct peanut shell as mineralized skeletons, finally mixed with PM to obtain adhesives.	The adhesive obtained had a strong water resistance, high bonding strength, and strong mold resistance, while it had a decrease in moisture absorption rate and viscosity	[110]
	Plant protein-based adhesives	Hot-pressed PM, sodium dodecyl sulfate (SDS), papain, urea, polyamide epichlorohydrin	Mixed hot-pressed PM with papain, urea and SDS at room temperature	Compared with hot-pressed meal adhesives, the wet shear strength increased 96.4%, the mass loss and moisture uptake value reduced by 41.4% and 69.4%, and the viscosity increased by 30.4%	[107]
Adhesives	Plant protein-based adhesives	Hot-pressed PM, SDS, nano-silica (nSiO_2_), polyamide polyamine epichlorohydrin (PAE)	Dissolved hot-pressed PM in 1.07% SDS, mixed at 60 °C, and added 37.3% PAE	The adhesive obtained was a tan opaque liquid, could penetrate deeply into the voids of the birch, could form hyperbranched cross-linked network structures with birch, and prevented moisture effectively	[38]
	Plant protein-based adhesives	Defatted PM, SM, triglycidylamine (Tga)	Incorporated Tga in plant meal solution, and stirred at room temperature	The wet and dry shear strength of the plywood bonded by resulted plant protein-based adhesive increased by 50% (1.02 ± 0.02 MPa) and 31% (1.55 ± 0.03 MPa)	[111]
	Plant protein-based adhesives	HPMP, polyamide polyamine, nSiO2, SDS, epichlorohydrin resin (PAE)	SDS, nSiO_2_, and PAE were added into the HPM solution in turn and stirred continuously at 60 °C	The obtained adhesive had a good boiling water strength (0.85 MPa), and an increase in water resistance, a good extension on mildew resistance for over 12 days, and the solids content and viscosity of the obtained adhesive increased by 45.4% and 274.6%	[112]
	Plant protein-based adhesives	PM, urea (U), epichlorohydrin (ECH)	Modified the defatted PM with U and ECH under 50 °C, during which the modified peanut was stirred several times in a three-neck flask equipped with a condenser	The decomposition temperature of the protein skeleton structure increased to 314 °C, improved the water resistance, and prevented the moisture penetration	[113]
Adhesives	Plant protein-based adhesives	Hot-pressed PM, SDS	Modified hot-pressed PM at 60 °C for 3 h, material:liquid = 1:3	The average value of the adhesive bonding strength is 1.05 ± 0.07 MPa	[114]
	Plant protein-based adhesives	PM, SDS, ethylene glycol diglycidyl ether (EDGE)	Modification time 3 h, material:liquid = 1:3	The viscosity of the adhesive obtained was 24,140 MPa/s, water resistance increased 10%	[115]
Films	Films	PM, citric acid, glycerol, alkaline solutions	Extracted peanut protein by alkaline solutions, and mixed with glycerol, then cast it on aluminum sheets and molded films by compression machine at 40,000 psi, 150–175 °C	The strengthening of the prepared films is 8.0 MPa, and the high elongation is 63%, modulus is 147 MPa	[116]
	Films	PM, citric acid, NaOH solution	Extracted peanut protein by NaOH and cast it with Teflon glass plates	The cross-linked peanut protein films exhibited good dry and wet strengths, with a film strength up to 6.1 MPa, with breaking elongation of 66%	[117]
